# Open‐Source Multinuclear Low‐Field Preclinical MRI Scanner

**DOI:** 10.1002/nbm.70291

**Published:** 2026-04-27

**Authors:** Gonzalo G. Rodriguez, Sergey Korchak, Charlotte von Petersdorff‐Campen, Oscar Sucre, Jan Felger, Ruhuai Mei, Stefan Glöggler

**Affiliations:** ^1^ NMR Signal Enhancement Group Max Planck Institute for Multidisciplinary Sciences Göttingen Germany; ^2^ Center for Biostructural Imaging of Neurodegeneration University Medical Center Göttingen Göttingen Germany; ^3^ Department of Biomedical Engineering University of Texas Southwestern Medical Center Dallas Texas USA

**Keywords:** hyperpolarization, low‐field, multinuclear MRI, open‐source, preclinical

## Abstract

Low‐field MRI (B_0_ ≤ 0.2 T) is emerging as a technology with the potential to revolutionize clinical diagnostics and patient monitoring. The reduction in magnetic field strength comes along with a decrease in the cost of the scanners and the infrastructure required for their operation. This, in turn, enables the development of compact, portable systems that extend imaging capabilities to locations and scenarios that were previously inaccessible. This democratization has empowered research groups worldwide to design and build their own scanners, resulting in a surge of new devices. However, the majority of low‐field scanners are optimized for human studies, which limits their application in preclinical research and constrains the testing and development of contrast agents tailored for low‐field regimes. In particular, hyperpolarized agents hold considerable promise for low‐field MRI due to their capacity to enhance signal and provide metabolic imaging of living systems. This signal enhancement counteracts the inherently low signal‐to‐noise ratio of thermally polarized studies, which is the main limitation of low‐field MRI. To exploit this potential, hyperpolarization studies frequently require the detection of X‐nuclei, such as ^13^C and ^15^N, thereby emphasizing the need for versatile, multinuclear preclinical scanners. In this study, we present an open‐source multinuclear low‐field MRI scanner that has been specifically designed for hyperpolarized preclinical research. The device operates at 66 mT and allows the detection of ^1^H, ^23^Na, ^13^C, and ^15^N nuclei. Comprehensive documentation and complete CAD designs are provided to facilitate replication and adaptation by the research community. To illustrate the performance of the system, the results of the acquisition of NMR spectra from each nucleus are presented, as well as the first images obtained with the scanner. This platform aims to bridge the gap in preclinical low‐field MRI, enabling rigorous in vivo testing of novel contrast agents and supporting broader innovation in the field.

## Graphical abstract

A step‐by‐step guideline for the construction of an open‐source multinuclear low‐field MRI scanner for preclinical studies is presented together with the first images acquired with the device. The scanner can detect ^1^H, ^23^Na, hyperpolarized ^13^C, and ^15^N signals at 66 mT.

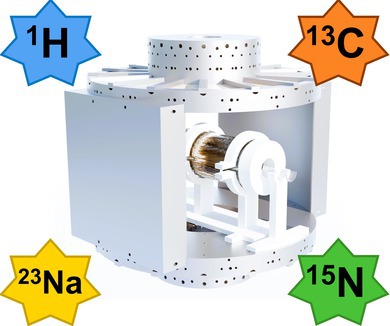

## 1 Introduction

Low‐field (B_0_ ≤ 0.2 T) magnetic resonance imaging (MRI) is emerging as a technology that promises to democratize access to MRI [1, 2, 3]. The reduction of the main magnetic field can drastically decrease the cost of the scanner, infrastructure requirements, size, and even enable portability [4, 5, 6, 7]. This makes possible the implementation of MRI under conditions and in places that were impossible before, such as ambulances [8] and intensive care units [9]. Furthermore, it has already demonstrated clinical potential for the detection and monitoring of several pathologies, i.e., stroke [10] and child brain development [11]. In addition to its tremendous clinical potential [12], low‐field MRI is reshaping the research landscape. The reduced costs of this technology have enabled numerous groups worldwide to enter the field, prompting a shift from specialization in software development or specific hardware components—such as gradient or RF coils—to the capability to design and build complete MRI systems. This increased flexibility has led to a flourishing diversity of scanner designs [7, 13], from devices based on electromagnets [14, 15], including field‐cycling systems [16, 17], to permanent magnets with Halbach [4, 6, 18], biplanar [19, 20, 21, 22], incorporated gradients [5, 23], and even single‐side designs [24, 25]. The rising interest in low‐field MRI has even led to the organization of hackathons, where students and researchers from around the world come together to build a scanner in just a few days [26, 27]. These events offer valuable educational opportunities, strengthen the scientific community, and foster collaboration. Because of this vibrant community and enhanced collaboration, numerous groups have begun to publicly share their designs, increasing reproducibility and extending the open‐source philosophy from software to hardware [28]. This trend is exemplified by initiatives such as the Open‐Source Imaging project (www.opensourceimaging.org) and is highlighted in this special issue. Particularly, the growing availability of open‐source resources for both hardware and software has been instrumental in advancing all stages of our developments, from scanner design to image acquisition, thereby accelerating progress. In this work, we utilized publicly available mathematical models for RF coil design [29, 30], open‐source software for gradient and shimming coil optimization [31, 32], and established algorithms for undersampling and image reconstruction [33]. These accessible tools facilitated rapid prototyping and implementation. They have also motivated us to openly share all of our developments with the research community to continue fostering reproducibility and collaborative development.

Despite the exponential growth in low‐field MRI research, most of the current reported devices are optimized for human studies, with few scanners dedicated to preclinical investigations in small animals [16, 34, 35, 36]. This does not only represent a limitation for low‐field preclinical research itself but also is relevant for the development of contrast agents that can be more efficient at low magnetic fields [35, 37, 38] or even impossible to implement at high fields [39, 40, 41]. Prior to the translation of these agents to human studies, it is imperative that their biocompatibility and performance are thoroughly evaluated preclinically. Consequently, the advancement of open‐source preclinical scanners holds the promise of propelling and expediting the development of next‐generation contrast agents tailored for low magnetic fields.

Among the available contrast agents, hyperpolarized compounds exhibit considerable potential for use in low‐field applications. Hyperpolarization is a technique that enhances nuclear magnetic resonance (NMR) signals by more than 4 orders of magnitude [42]. Therefore, given that this enhanced polarization is not dependent on the detection B_0_ field, the utilization of hyperpolarized agents has the potential to address a significant constraint of low‐field MRI: the suboptimal signal‐to‐noise ratio (SNR) resulting from the limited ^1^H thermal polarization [43]. In hyperpolarization experiments, the signal enhancement of nuclei with low gyromagnetic ratios such as ^13^C and ^15^N is typically targeted. The main motivation for using nuclei with low gyromagnetic ratio is to store the magnetization for longer times, given that these nuclei usually exhibit longer T_1_ and this determines the decay of the hyperpolarized signal. The signal enhancements produced by hyperpolarization have enabled the measurement of real‐time metabolism in vivo [44, 45] and are considered to be the next‐generation molecular imaging tool for tumor grading and therapy response assessment [46, 47]. Several ^13^C [44, 46, 47, 48, 49] and ^15^N [50, 51, 52, 53, 54] hyperpolarized contrast agents have been intensively investigated at high magnetic fields (B_0_ ≥ 3 T). Nevertheless, despite the great potential of hyperpolarization for low‐field applications, there are only few studies reported [36, 55, 56, 57] in the milli‐T regime (1 mT ≤ B_0_ ≤ 200 mT). A major limitation for expanding these investigations is the almost nonexistence of low‐field multinuclear MRI scanners capable of detecting ^13^C and ^15^N [55, 58, 59]. In addition to the multinuclear capability, the low gyromagnetic ratio of ^13^C and ^15^N requires the development of receive coils that maximize the filling factor to increase SNR [29] and high gradient amplitudes for k‐space encoding. In this regard, the current low‐field scanners typically show RF coils for human brain or extremities, with a very low filling factor for mice and gradients with efficiency below 1 mT m^−1^ A^−1^ [6, 18].

In this work, we introduce our open‐source multinuclear low‐field preclinical scanner capable of detecting ^1^H, ^23^Na, ^13^C, and ^15^N signals at 66 mT. In a recent study, we successfully measured the conversion of hyperpolarized ^13^C pyruvate to lactate in cancer cells with this device [55], demonstrating the potential of this field regime for real‐time metabolic monitoring. Additionally, we recently monitored a ^15^N chemical reaction in real time for the first time in the mT regime [60]. Here, we present a comprehensive description of the design, construction, and assembly of the main magnet, gradient, shimming, and RF coils. Moreover, we show NMR signals acquired from ^1^H and ^23^Na thermally polarized phantoms and hyperpolarized ^13^C and ^15^N samples. Finally, we present MRI images with hyperpolarized ^13^C, together with images for ^1^H and ^23^Na nuclei, all of them acquired with our scanner.

## 2 Multinuclear Low‐Field Preclinical Scanner

**FIGURE 1:**
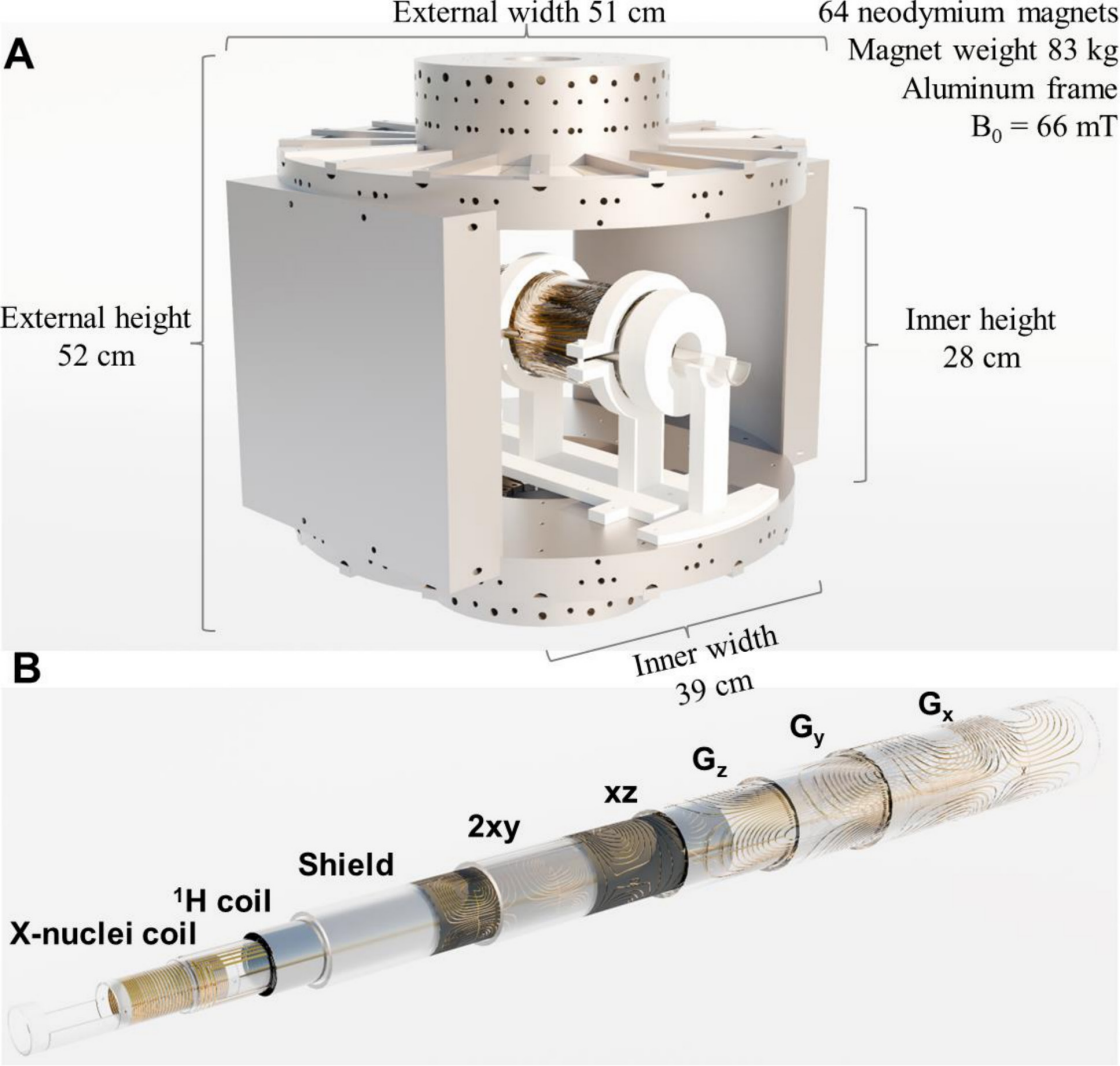
(A) Render of the low‐field multinuclear scanner. (B) Telescopic view of the RF, shield, and shimming coils.

The scanner was designed for preclinical studies of small animals with a maximum active volume of a 90‐mm length and a 36‐mm diameter, which is enough for mice experiments. The device has three linear gradient coils employed for both shimming and spatial encoding, two additional second‐order shimming coils, and two transmit/receive RF coils, one used for ^1^H excitation and detection and the other one for the X‐nuclei (^23^Na, ^13^C, and ^15^N). For most of our experiments, the ^23^Na channel is mainly used for calibrating the ^13^C pulses and sequences without the need of using hyperpolarized samples (due to the close gyromagnetic ratio between ^23^Na and ^13^C). The RF coil has an incorporated bed to facilitate the positioning of the mouse and increase animal comfort. All these components, together with the supports for the assembly of all the pieces, were designed and built in‐house. The CAD files for all the parts are publicly available in STL and STEP formats on GitHub. Figure 1 shows the render of our scanner together with a telescopic view showing all the elements. All the electronics were purchased from commercial vendors. Specifically, we use a Tecmag Bluestone LF2 MRI console (Houston, United States) with two BT00250‐AlphaS Tomco (Adelaide, Australia) RF amplifiers. The linear gradient coils are connected to three AE Techron 2110 amplifiers (Elkhart, United States) and a six‐channel shimming power supply MXB‐6‐RTC, Resonance Research Inc. (Billerica, United States).

## 3 Magnet

**FIGURE 2:**
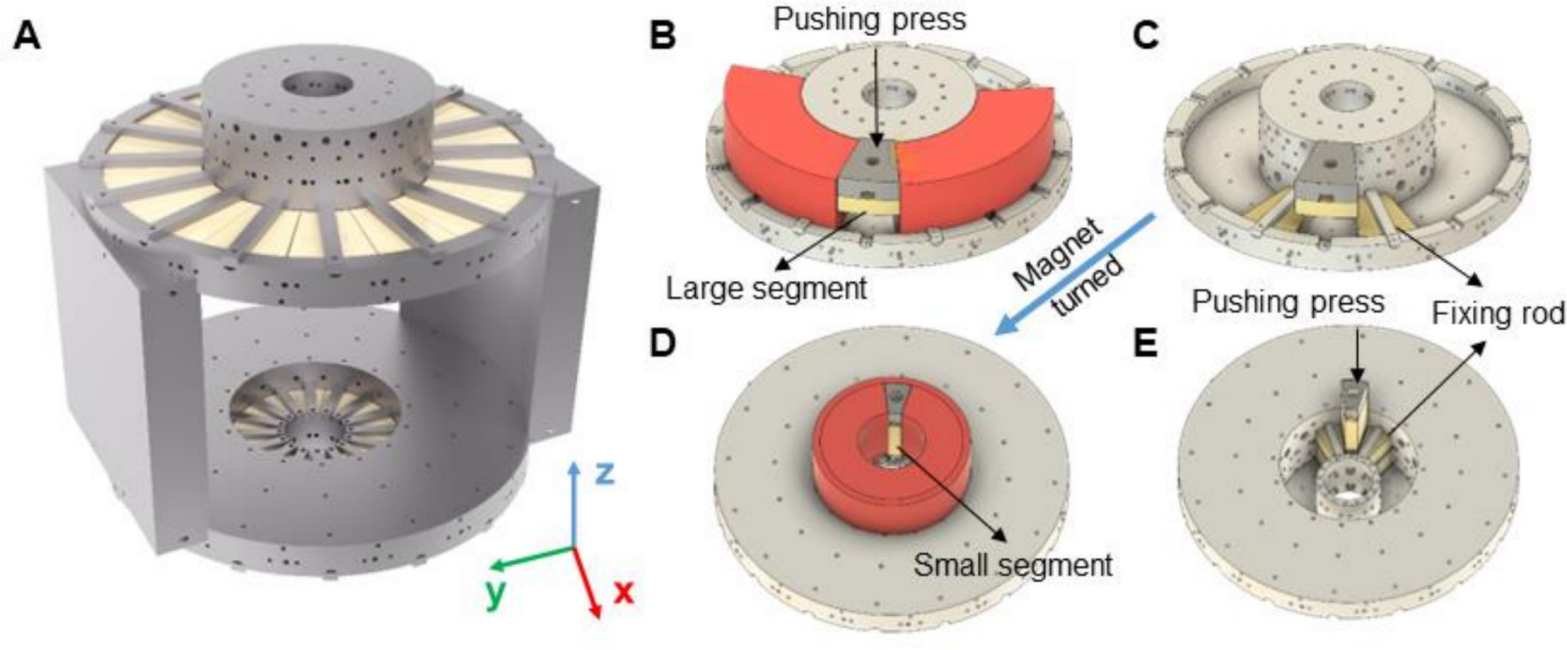
(A) Render of the complete assembled magnet. (B, C) Large ring magnet segment placement. (D, E) Small ring magnet segment placement. The pushing press is shown in dark gray, and the 3D‐printed plastic guiding tunnels are shown in red.

The magnet was designed with portability, lightweight construction, and versatility in mind, ensuring sufficient space for the incorporation of shimming coils to achieve the spectral resolution necessary for measuring pyruvate‐to‐lactate conversion. To meet these requirements, we implemented an optimization method based on the spherical harmonics description of the magnetic field, which allowed us to find the precise dimensions of an array of four axially magnetized rings [61]. This array with axial and mirror symmetry is such that the zonal spherical moments are balanced out up to 7 orders. This magnet features a 28‐cm gap, providing ample room for shimming coils and future expansion to large animals or even clinical studies. The magnet is composed of two sides, each of which consists of 32 neodymium magnets arranged in two different shapes (16 each). Shape 1 consists of a 22° segment (inner radius 47.9 mm, outer radius 100.0 mm, 71.0 mm height), and Shape 2 is a 22° segment (inner radius 121.7 mm, outer radius 225.0 mm, 25.0 mm height). Constructed with an aluminum frame, the final magnet weighs only 83 kg, making it one of the lightest portable designs currently available [13, 26]. Figure 2A shows the render of the fully assembled magnet. The magnets were purchased from OSENC Company (Dongguan Osenc Magnet Co., LTD, China) via https://osenc.com/, with a total cost of $11,304 in 2022 and a less than 3 months delivery time. The aluminum frame was turned in a 5‐axis CNC milling machine (DMU 65 monoblock).

Magnetic field simulations were implemented in COMSOL Multiphysics 6.1 (Sweden) for a field of view defined by a cylinder of 100‐mm length and 40‐mm diameter placed in the geometrical center of the magnet, which represents the maximum ROI of the current design. The simulated field in the center of the magnet was 67.96 mT, and a peak‐to‐peak inhomogeneity in the full cylinder volume without the incorporation of the shimming coils was 2796 ppm, with the main inhomogeneity component along the x direction. After construction, the measured magnetic field in the geometrical center was 66 mT, and the inhomogeneity measured as the full width of the NMR spectrum of a cylindrical water phantom of 57‐mm length and 21‐mm diameter was 1782 ppm, with a full width at half maximum (FWHM) of 766 ppm [62]. Whereas the simulations over the same volume showed a peak‐to‐peak inhomogeneity of 486 ppm, after the implementation of the active shimming, the magnetic field at the center was 66 mT and the inhomogeneity was 56 ppm (FWHM). It is worth noticing that the experimental magnetic field at the geometrical center differs by less than 3% with respect to the simulated theoretical value, whereas the inhomogeneity shows a deviation of 3.6 times with respect to the simulations, which are within the expected range taking into consideration other neodymium magnet designs [26]. Below, we describe the magnet assembly procedure.

### 3.1 Magnet Montage Procedure

1 Simulation results

COMSOL simulations of assembly forces were conducted. Results indicate that the large ring segments should be assembled first, followed by the smaller ones. During assembly, new segments are pulled toward the center and onto previously installed segments, while also being pushed slightly out of their final position. This displacement requires the use of a pushing mechanism for proper installation.

2 Guiding tunnel

A tunnel made from 3D‐printed plastic forms can be used to safely guide the segments, restricting their movement to the vertical direction only (Figure 2B, guiding tunnel in red).

3 Initial segment installation

The first two segments can be safely installed in positions that leave a void between them. Round rods were temporarily placed on both sides to prevent unwanted horizontal shifting.

4 Filling the void
  ○ The third segment is inserted into the void between the first two (Figure 2C; guiding tunnel is not shown for better visualization).
  ○ Magnetic forces push this segment upward and sideways onto the existing ones, while pulling the first two segments horizontally toward the void.
  ○ The first two segments are held in place with fixing rods. Additional temporary rods prevent horizontal movement.
  ○ The third segment is pushed downward within the guiding tunnel by using the pressing machine (dark gray in Figure 2) until it reaches the temporary rods. These rods are then removed, allowing the segment to be fully pressed into position.
  ○ A square fixing rod is installed through an opening in the pushing foot to secure the segment.
5 Repeating the process
  ○ The fourth segment is installed adjacent to the first three, again leaving a void space.
  ○ It is secured with permanent fixing rods and temporary round rods.
  ○ The void is then filled by the next segment, following the same procedure.
  ○ This sequence is repeated until all large segments are in place.
6 Installation of small segments
  ○ The magnet is turned upside down to begin assembly of the smaller segments (Figure 2D,E).
  ○ The process is similar to that for the large segments:
    - The first two small segments are installed with a void between them, stabilized by four temporary round rods per segment
    - The third segment is inserted into the void using the pressing machine until it reaches the temporary rods. The rods are removed and the segment is pressed into position.
    - The fourth segment is added next to the existing three, again leaving a void to be filled by the next segment.
  ○ The sequence is repeated until all small segments are installed.
7 Final stand assembly
  ○ Once both sides of the magnet are fully assembled, support stands can be added.
  ○ Two stands are mounted on one side and fixed securely with screws.
  ○ For safety, the gap between the two sides is filled with an appropriate material to reduce risk during handling.
  ○ The second side of the magnet is then carefully aligned, brought into position, and fastened with screws.
  ○ Interestingly, the attraction force between the two completed sides is found to be relatively small, allowing for safe final assembly.

## 4 Gradient and Shimming Coils

Three linear gradient coils (G_x_, G_y_, and G_z_) and two second‐order shimming coils (2xy and xz) with cylindrical geometry were designed and built in‐house. The linear coils are used for active shimming, as well as for spatial encoding during image acquisition. The selection of the 2xy and xz coils was based on the B_0_ magnetic field simulations that showed the main component of inhomogeneity along the x‐axis. The G_y_ and G_z_ coils were designed with our own software based on a target field method reported in the literature [32]. For the G_x_, 2xy, and xz, we decided to migrate to the open‐source CoilGen software [31]. CoilGen offers several improvements as it takes into account the size of the wires and the crossing between paths. Both target field and CoilGen algorithms were implemented on Matlab 2023b. The shimming and gradient coils were designed to be as close as possible to the sample in order to increase the coil efficiency as the low gyromagnetic ratios of ^13^C and ^15^N require the implementation of strong gradients for imaging. This resulted in a compact assembly, as shown in Figure 1B. It is worth noting that, as the hyperpolarization experiments involve single‐shot and fast acquisitions, there is no need to incorporate a cooling system. Nevertheless, the thermal stability of the system should be taken into account if long acquisitions for proton images are required. Experimentally, after 1 h of running our most gradient‐intensive pulse sequence (RARE sequence with 32 echoes and 400‐ms TR), we did not require the implementation of a cooling system with measured thermal drifts below 48 Hz/min for ^1^H that did not generate observable artifacts in the image [63]. All the gradient coils showed less than 5% deviation within the region of interest (a cylinder with 46‐mm diameter and 90‐mm length) from a perfect linear response, showing good agreement between the simulation and experimental measurements. The G_x_ showed a deviation of 5% with respect to the targeted gradient efficiency, whereas the G_y_ and G_z_ showed an 8% deviation. This suggests that CoilGen is more reliable than our target field approach. Table 1 shows the geometrical and electrical characteristics of the five coils built. Below, we describe our step‐by‐step procedure for the construction of the coils. Figure 1B shows the render for all the gradient and shimming coils.

**TABLE 1:** Geometrical and electrical parameters of each gradient and shimming coil.

	G_x_	G_y_	G_z_	xz	2xy
Diameter (mm)	100	90	80	68	64
Length (mm)	314	314	314	150	150
Wire section (mm)	0.8	0.8	0.8	0.5	0.5
Efficiency (mT m^−1^ A^−1^)	1.05	2.25	2.79	—	—
Resistance (Ω)	0.7	0.3	0.3	0.7	1.0
Inductance (μH)	43	14	12	160	120

### 4.1 Step‐by‐Step Gradient and Shimming Coil Construction

**FIGURE 3:**
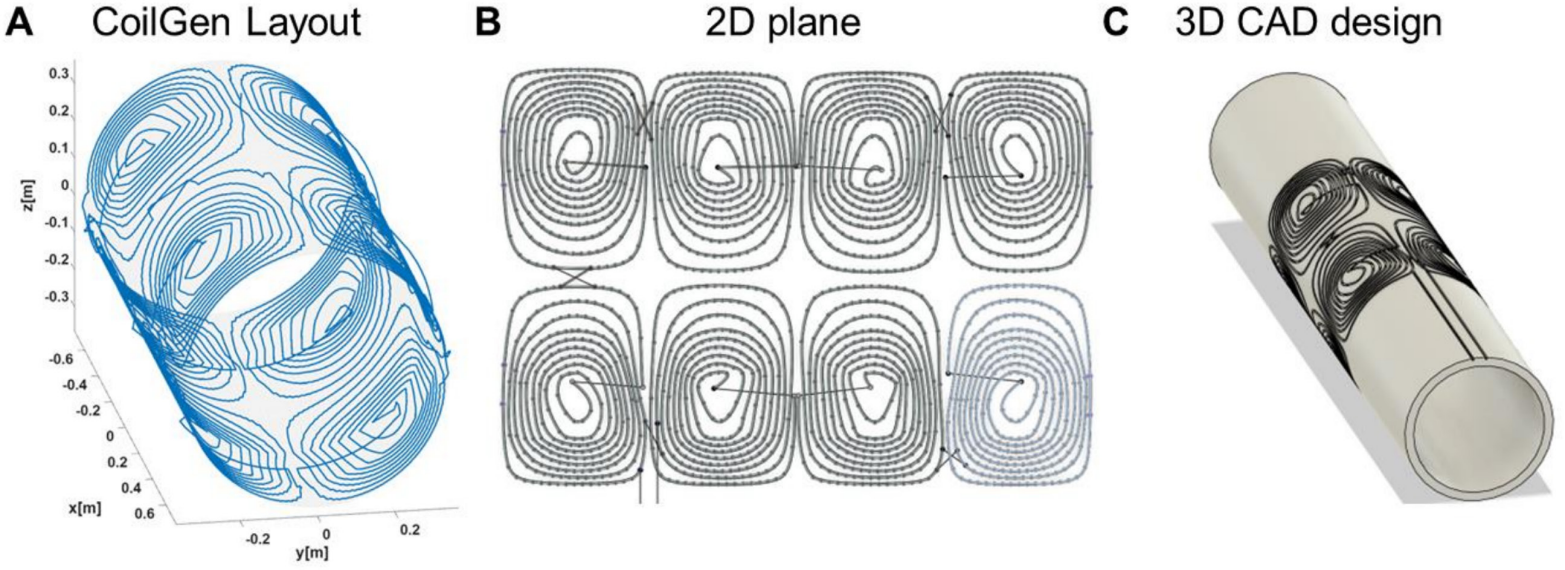
Step‐by‐step 2xy shimming coil design. (A) 3D layout obtained from CoilGen. (B) Unfolded 2D plane with incorporated connections between individual loops (solid straight lines). (C) Final 3D solid CAD design after folding the 2D plane into a solid cylinder by using the “Emboss” function in Autodesk Fusion 360.

1 Coil layout generation
  ○ Mesh for coil surface (for us a cylindrical geometry) and mesh for the region of interest for the generated magnetic field are defined (for us a cylinder with 46‐mm diameter and 90‐mm length).
  ○ Wire size and desired target field are defined.
  ○ Mesh is imported and a dedicated software is used to generate the coil layout (target field method or CoilGen).
  ○ Coil layout is exported as a 3D trajectory (Figure 3A).
2 From layout to STL file

Directly importing the 3D coil layout to a CAD design software resulted in errors that did not allow us to generate a 3D model for 3D‐printing or CNC machines. Therefore, we follow the steps described below to be able to avoid all the errors:

○ The 3D layout trajectory (Figure 3A) is unfolded from the cylinder surface to a 2D plane (Figure 3B).
○ Individual loops are selected from the 2D plane and imported one by one in our CAD software (Autodesk Fusion 360).
○ Each loop is converted to a 3D body using the “Pipe” function with the specific shape and diameter of the desired copper wire.
○ Connections between loops are done manually (solid black lines in Figure 3B) trying to follow the original paths from CoilGen.
○ The 2D body is folded back to its original cylindrical shape and extruded with a cylinder with the desired inner and outer diameters (by using the “Emboss” function), resulting in the final 3D body CAD file (Figure 3C).

3 Construction
  ○ The wire paths for the linear coils are machined with a CNC device in acrylic glass (PMMA), whereas the 2xy and xz coils were 3D printed with Formlabs Grey Resin V4 (Somerville, United States).
  ○ The wires are manually placed through the paths and glued with superglue.

## 5 RF Coils

**FIGURE 4:**
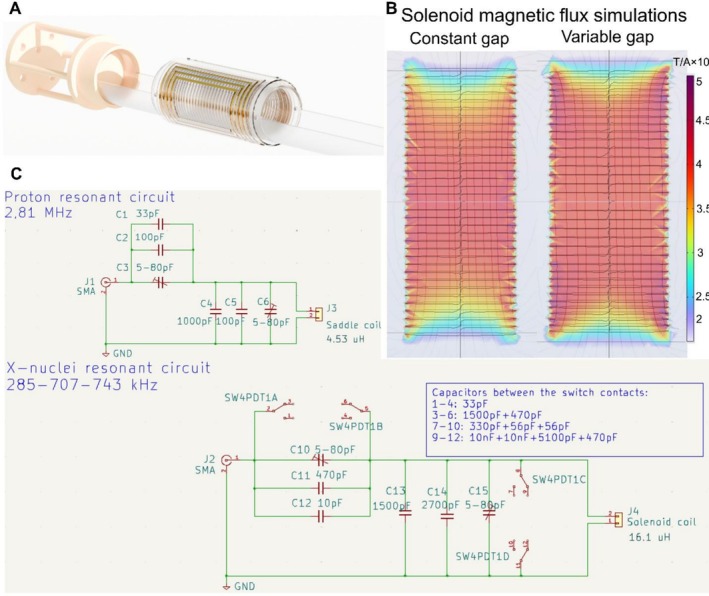
(A) Render of multinuclear RF coils; the saddle (outer) coil is used for ^1^H detection, and the solenoid (inner) is used for X‐nuclei. (B) Magnetic field COMSOL simulations of solenoids with constant vs. variable gap. (C) Schematic of resonant circuits.

With the purpose of having them electrically decoupled, the concentric RF coils for the ^1^H and X‐nuclei are made following the saddle and solenoid designs, respectively. The saddle coil has 5 loops with a diameter of 46 mm and a length of 90 mm. The solenoid has 35 loops with a gap of 3 mm for the 15 center loops, a gap of 2.5 mm for the 5 consecutive loops, and a gap of 2 mm for the 5 outer loops to improve homogeneity. The diameter of the solenoid is 40 mm and the length is 90 mm. The coils were empirically optimized and wound with Litz wires with 60 filaments of 0.1 mm^2^ (reichelt.de) to increase the Q values and improve SNR [43]. The saddle coil was dedicated to ^1^H detection, whereas the solenoid was dedicated to X‐nuclei detection. For selecting the resonance frequencies of the different nuclei, an analog switch was incorporated into the RLC circuit in order to select different matching and tuning capacitors. In addition to the switch, variable capacitors were incorporated to perform fine‐tuning and matching adjustments for each resonance frequency. The coils were mounted in a cylindrical acrylic tube with a mouse bed incorporated to facilitate future preclinical experiments. Figure 4A shows the render of the complete RF multinuclear coil. The capacitors for each resonant circuit were soldered onto a PCB board placed in the white structure shown in Figure 4A. The variable capacitors for the fine‐tuning, the analog switch for the X‐nuclei selection, and the SMA coaxial connectors are accessible from the back of the coil. Figure 4B shows the B_1_ maps simulated in COMSOL for a solenoid with 35 loops and with a constant pitch gap of 2.57 mm and the proposed design with variable gap. The homogeneity of the B_1_ in the extremes of the coil shows clear improvements when compared with the constant gap. Figure 4C shows the proton and X‐nuclei resonant circuits. The coils showed Q values of 147, 260, 240, and 160 at the resonance frequencies of the ^1^H, ^23^Na, ^13^C, and ^15^N nuclei, measured as the resonance frequency divided by the resonator bandwidth (FWHM) obtained with a network analyzer.

### 5.1 RF Coil Design and Construction

1. Both coil designs are empirically optimized following the guidelines reported in the literature [29, 30].
2. Given the simple geometry of the coil designs, the STL and STEP files required for the construction are manually done in Fusion 360.
3. The paths for wires in each coil are milled on an acrylic glass (PMMA) tube. Nevertheless, they can be 3D‐printed.
4. The wires are manually placed into the paths and glued with epoxy “superglue”.
5. The saddle coil is attached to the solenoid with plastic screws.

## 6 Coil Assembly and Mounting

**FIGURE 5:**
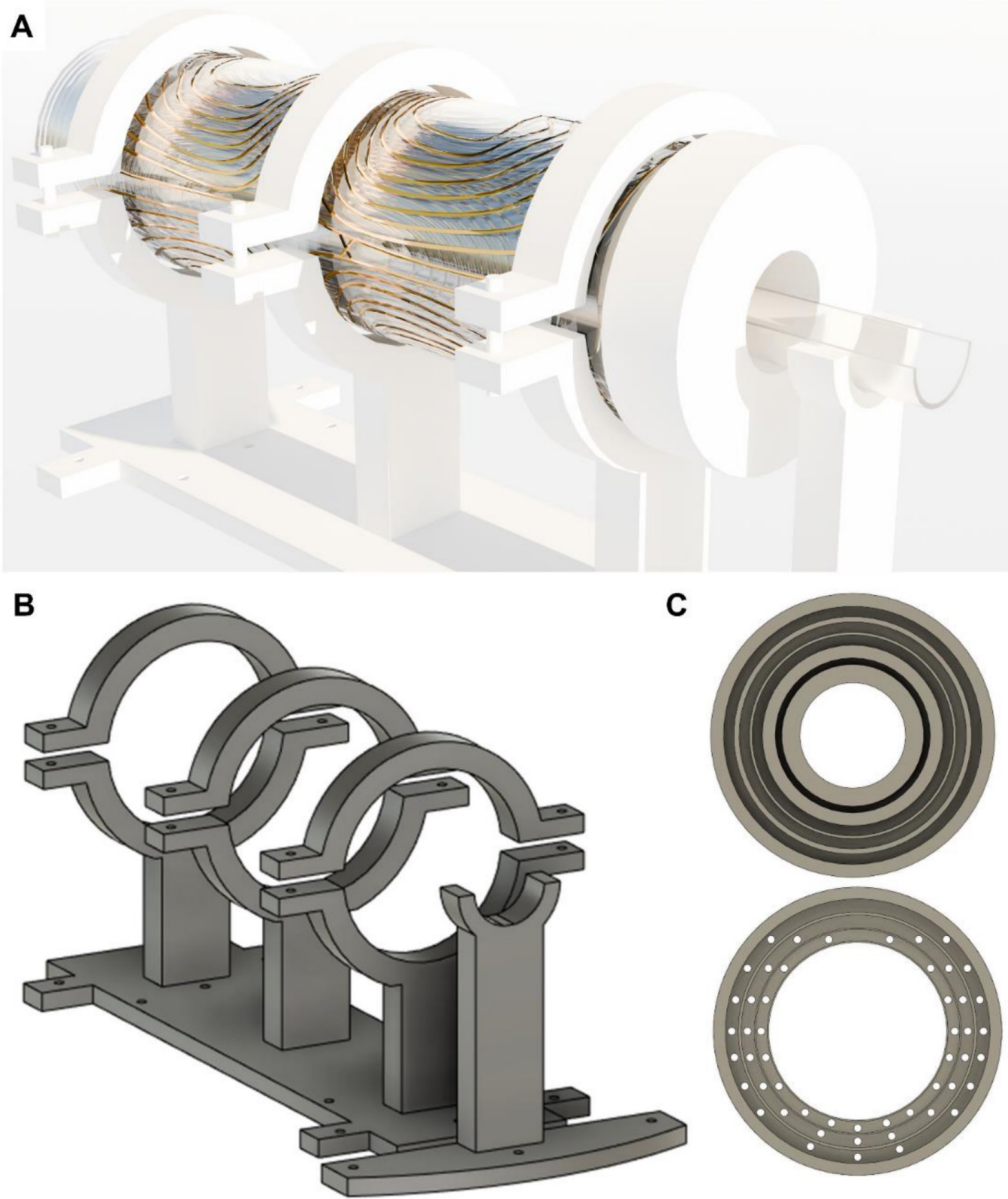
(A) Render of the assembled coils setup and its support. (B) Coil support. (C) Pieces for fixing coils positioning and centering them. The top element is placed on the right side of the coils and fixes the five shimming coils, the aluminum shield, and RF coils. The bottom element is placed on the left and fixes the gradient coils. The holes were done to allow the exit of the gradient cables.

Following the construction of all the coils, several additional components were designed to guarantee the correct and stable position. Figure 5A shows the render of the final assembly of the coils setup. Figure 5B shows the design of the coil supports, which were screwed to the bottom face of the magnet. Then, two ending caps were built in order to keep the coils centered and fixed. The cap from the right part fixed all the components (top part of Figure 5C), and the cap from the left (bottom part of Figure 5C) only fixed the gradient coils in order to allow the removal of the RF coils. All the coil supports and fixing components are manufactured from POM (polyoxymethylene).

## 7 Multinuclear NMR Spectra and Images

### 7.1 NMR Spectra

**FIGURE 6:**
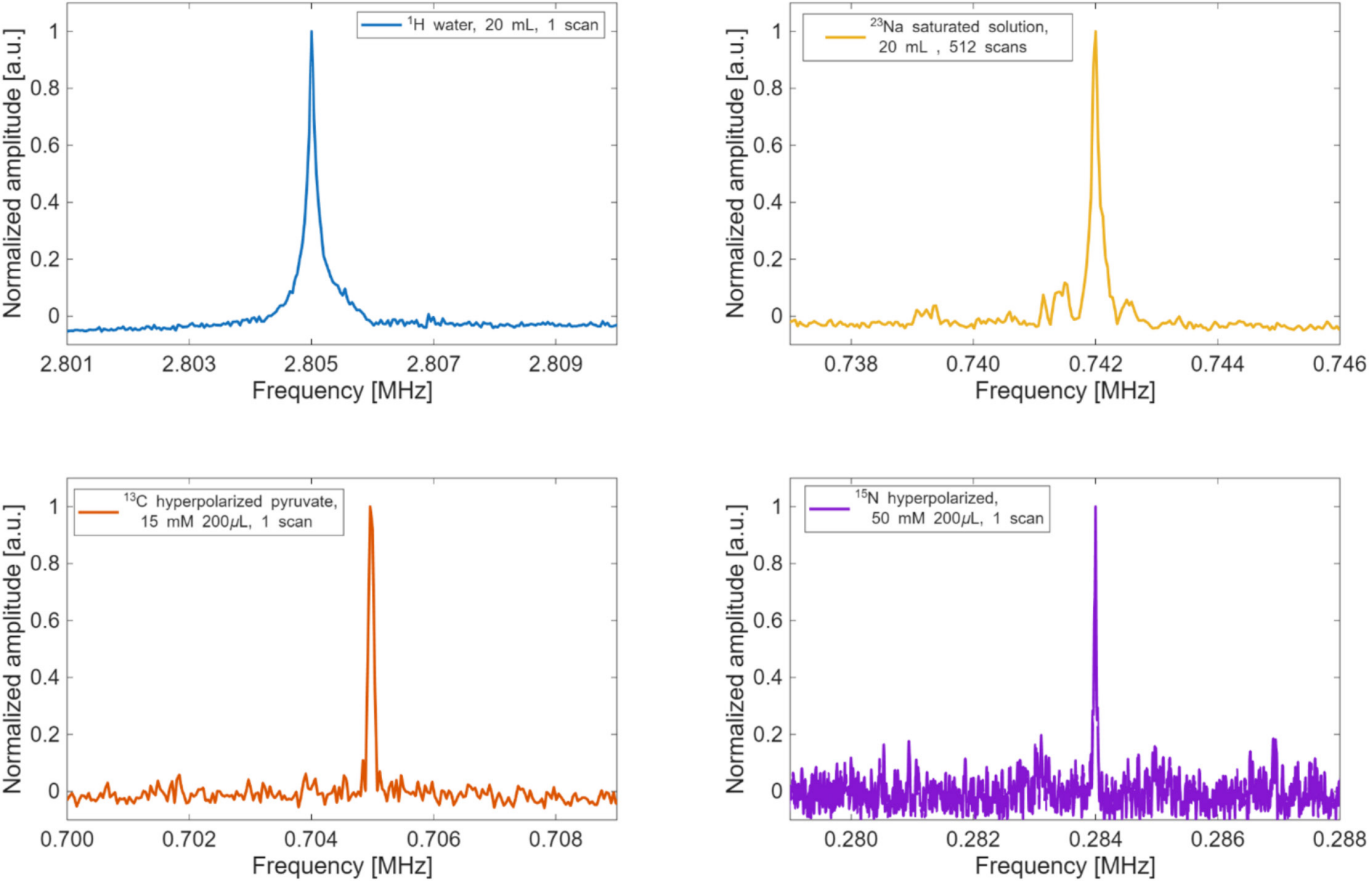
Normalized NMR spectra acquired for each nucleus. Blue: spectrum from 1 scan acquired from a 20 mL sample of water doped with 15 mM copper sulfate. Yellow: spectrum from 512 scans of a 20 mL sample of water saturated with sodium chloride. Orange: spectrum from 1 scan of 200 μL of a 15 mM hyperpolarized [1‐^13^C] pyruvate in D_2_O. Violet: spectrum from 1 scan of 200 μL of 50 mM ^15^N‐BBPEP.

Spin‐echo pulse sequences were implemented for signal detection in order to start the acquisition windows far from the coil ringing, which is critical at such low frequencies. The ^1^H NMR signal was detected over one scan of a 20‐mL phantom of water doped with 15 mM copper sulfate, using a spin‐echo sequence with echo time TE = 1.38 ms, acquisition time of 25.6 ms, and 256 acquisition points. The ^23^Na signal was detected over a 20 mL phantom with water saturated with sodium chloride after 512 scans, using a spin‐echo sequence with TE = 3 ms, acquisition windows of 10.24 ms, and 128 points. The ^13^C and ^15^N signals were acquired with the same sequence parameter with TE = 6 ms, acquisition windows of 102.4 ms, and 1024 points, after properly adjusting the flip angles for each nucleus (90° for ^23^Na and ^13^C with 32 μs length and ^15^N with 80 μs). The ^13^C signal was detected after a single scan of 200 μL of a 15 mM solution of hyperpolarized [1‐^13^C]pyruvate in D_2_O. The ^15^N signal was detected over 200 μL of a 50 mM solution of hyperpolarized ^15^N‐boronobenzyl‐2‐phenylethynylpyridinium (^15^N‐BBPEP) in MeOD developed in our lab [51]. Para‐hydrogen–induced polarization was used for all the hyperpolarization experiments [64, 65]. Pyruvate was hyperpolarized according to our previously published procedures [49, 66]. Figure 6 shows the spectra for all the nuclei. Once we successfully detected NMR signals for each nucleus, we proceeded to acquire images of ^1^H, ^23^Na, and hyperpolarized ^13^C phantoms.

### 7.2 Multinuclear images

**FIGURE 7:**
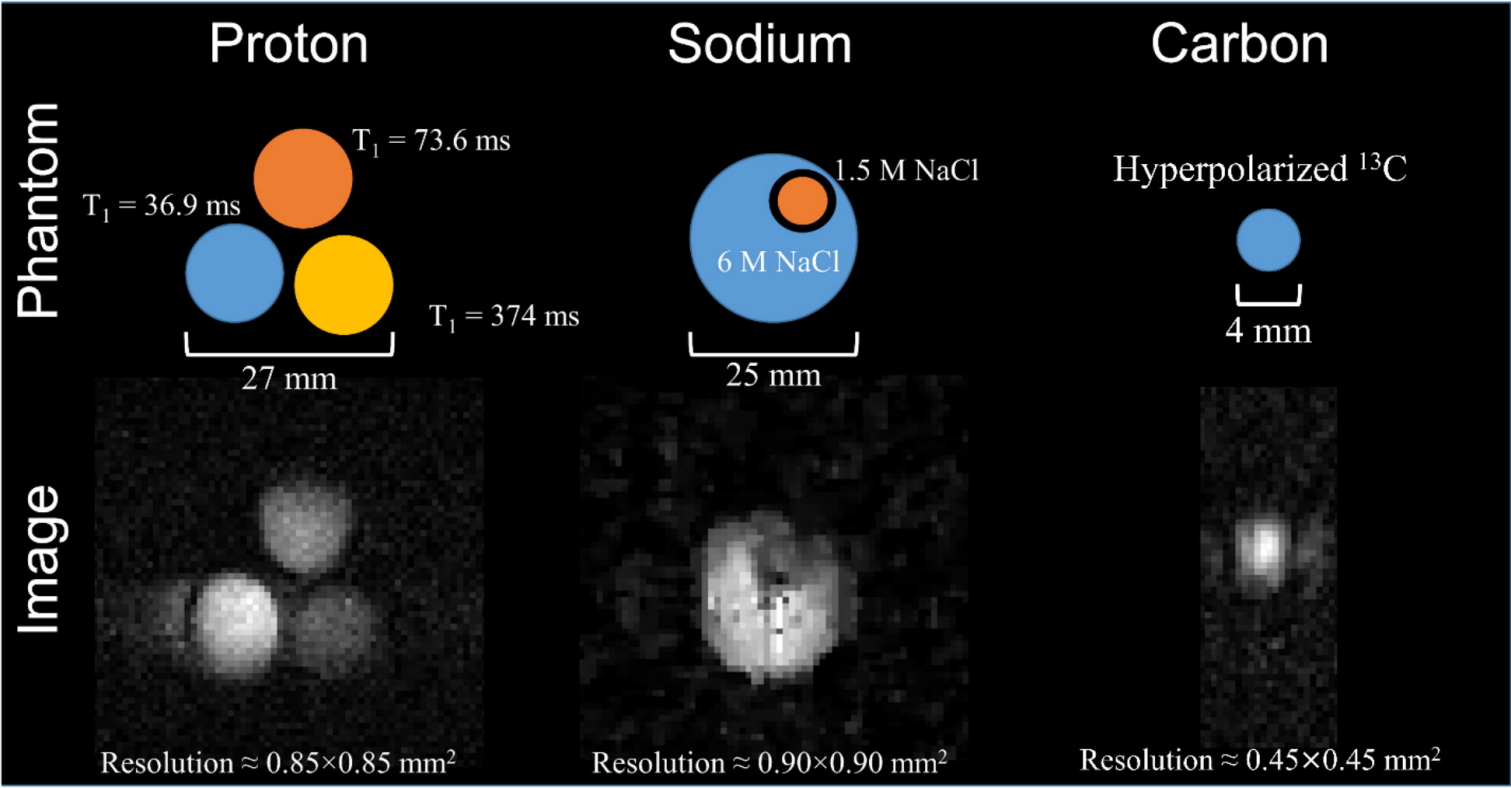
Multinuclear MRI at 66 mT. Left: proton image. Middle: sodium image. Right: carbon image. The top row shows the scanned phantom geometry with their main properties. The approximate proton resolution was 0.85 × 0.85 mm^2^, the sodium resolution was 0.90 × 0.90 mm^2^, and for carbon, it was 0.45 × 0.45 mm^2^.

All the images were acquired using spin‐echo–based sequences because they are more robust against inhomogeneity and instabilities generated by thermal drifts [63, 67]. For proton imaging, we implemented a fully sampled 2D spin‐echo sequence with TE = 2.8 ms and TR = 73.5 ms, 64 × 64 matrix, 128 averages, slice thickness 45 mm, and total acquisition time of 10 min. The scanned phantom was composed of three vials of 4 mL filled with deionized water doped with different concentrations of copper sulfate (15, 8, and 1 mM). The resulting T_1_ values for each sample were 36.9 ± 1.8, 73.6 ± 1.0, and 374 ± 14 ms, respectively. The proton image was reconstructed simply by implementing the 2D Fourier transform in Matlab R2023b. For sodium imaging, as a higher number of averages is needed due to the lower SNR, we implemented an undersampling factor of four along the phase‐encoding direction to keep the acquisition time < 1 h. The resulting pulse sequence was a 2D spin‐echo with TE = 5.8 ms and TR = 57.6 ms, 64 × 64 matrix, 2048 averages, slice thickness 60 mm, and total acquisition time of 31.5 min. The phantom was composed of a 20 mL vial filled with water saturated with NaCl (6 M) and another cylinder with 1.5 M NaCl. The image was reconstructed using a compressed sensing [68, 69] method publicly available (https://github.com/LudgerS/CSreconstruction) and previously reported for X‐nuclei reconstruction [33]. Additionally, to remove noise and increase smoothness, we applied a Gaussian filter in k‐space and removed the outliers in the image using the “filloutliers” (with the clip and quartiles methods) function from Matlab. For ^13^C imaging, the pyruvate precursor molecule 3‐(phenyl‐*d*_5_)prop‐2‐yn‐1‐yl‐1‐^13^C‐1,1‐*d*_2_ 2‐oxopropanoate‐1‐^13^C was hyperpolarized for a sample volume of 200 μL and a concentration of 21 mM as described by Ding et al. [70], with approximately 17% polarization on the ^13^C nucleus of the pyruvate moiety. The imaging sequence consisted of a 1‐scan 2D RARE sequence [71] with 32 echoes. The echo time was 3.1 ms and the phase‐encoding amplitude increases with the number of echoes to maximize SNR. The odd echoes were reconstructed due to phase cancelation on even echoes [4]. The image was reconstructed with NUFFT and zero filled to a 64 × 24 matrix to obtain a similar resolution in both encoding directions. Figure 7 shows the built phantoms for each nucleus together with the image acquisition.

## 8 Discussion

We presented a multinuclear low‐field scanner developed for preclinical studies. The CAD designs for all the built components and specific tools are publicly available, together with the COMSOL simulations for the B_0_ and B_1_ fields generated by the main magnet and the X‐nuclei coils, respectively. During the construction, we took advantage of the facilities and expertise from the fine mechanics workshop at our institution. Therefore, many of the components were constructed using CNC machines and were performed by dedicated technicians. Nevertheless, all the parts, except the aluminum frame, can be built by using a 3D printer as exemplified here for the shimming coils. This will simplify the construction and reduce the costs.

As an alternative to the commercial software COMSOL, the magnetic field simulations can be implemented in open‐source software such as Elmer FEM. Similarly, the gradient and shimming coils can be simulated using pyCoilGen, which is the equivalent to the CoilGen software used here but for Python.

The integration of shimming coils provides our scanner with substantial flexibility, enabling acquisitions under varying magnetic field homogeneities and allowing experimental questions to be addressed that would otherwise be difficult to explore. In the context of contrast agent design for detecting metabolic conversions, a critical consideration is determining the minimum field homogeneity necessary to achieve the required spectral resolution. Equally important is assessing the robustness of pulse sequences to inhomogeneities and developing postprocessing methods to correct field‐induced distortions. Combined with its multinuclear capability and coils tailored for small animal imaging, this feature represents a key advantage of our device over most recently published low‐field scanners [7].

The design of the RF coils optimized for mice experiments maximizes the filling factor and therefore the SNR, when compared with coils developed for human experiments. Moreover, reducing the size of the gradient coils, i.e., optimizing them for mice instead of humans, allows us to approximately double the gradient efficiency compared with recently developed human low‐field scanners [6, 18].

We demonstrated the feasibility of acquiring NMR spectra from ^1^H, ^23^Na, and hyperpolarized ^13^C and ^15^N with our scanner. Moreover, we successfully acquired submillimeter multinuclear images at 66 mT in a scanner built in‐house. This demonstrates the potential of our device for the study of hyperpolarized contrast agents specifically targeted for low‐field applications. We implemented the simplest acquisition sequences and reconstruction methods to allow for a straightforward evaluation of the hardware performance. Nevertheless, higher image quality can be achieved by implementing turbo spin‐echo sequences and complex reconstruction and postprocessing methods, such as deep learning reconstruction [72] or super‐resolution algorithms [73].

The proton image shows a pure T_1_ contrast given that all the samples have the same proton density. The lower signals are associated with the longer T_1_ values, consistent with our short TR = 70 ms. For the sodium image, there are also visible contrast differences between the high‐ and low‐NaCl concentration samples; nevertheless, in this case, the resulting contrast is weighted by T_1_, T_2_, and sodium density. T_1_ and T_2_ are shorter for higher concentrations, explaining the lower contrast than the concentration ratio between the two samples.

The growing availability of open‐source resources for both hardware and software has been instrumental in advancing all stages of our multinuclear low‐field imaging, from scanner design to image acquisition, thereby accelerating our progress in the field. Specifically, we utilized publicly available mathematical models for RF coil design [29, 30], open‐source software for gradient and shimming coil optimization [31, 32], and established algorithms for undersampling and image reconstruction [33].

## 9 Conclusion

We presented a systematic guideline to reproduce our low‐field multinuclear scanner developed for preclinical imaging and spectroscopy. As far as we are aware, this represents the first multinuclear low‐field scanner specifically developed to be capable of mouse studies. We demonstrated that it is possible to acquire submillimeter multinuclear images with our device. Moreover, we believe that the access to our design will foster the in vivo investigation of novel contrast agents at low magnetic fields. Specifically, we foresee a great potential for the development of hyperpolarized probes specifically engineered for low‐field applications. This has the potential to enable metabolic imaging in low‐cost and portable setups and to make this technology widely applicable.

## Author Contributions

G.G.R. and S.K. contributed equally to this work. G.G.R., S.K., J.F., and O.S. designed and constructed the hardware; G.G.R. developed the software; G.G.R., S.K., and C.v.P.‐C. optimized the hyperpolarization protocol; G.G.R. and C.v.P.‐C. performed the experiments; R.M. synthesized the precursors; G.G.R., S.K., and S.G. wrote the paper; and S.G. conceived the project. All authors revised the manuscript.

## Funding

This study was supported by the Marie Curie Action (101150649), the European Research Council (949180), Max‐Planck‐Gesellschaft, and Bosch‐Forschungsstiftung.

## Conflicts of Interest

The authors declare no conflicts of interest.

## Data Availability

The data that support the findings of this study are openly available in GitHub at https://github.com/gonggr/Open‐source‐Multinuclear‐Low‐field‐Pre‐clinical‐MRI‐Scanner. This includes the STL and STEP files from all the components of the scanner and the RLC circuits used for the multinuclear resonator.
